# Deep learning model improves tumor-infiltrating lymphocyte evaluation and therapeutic response prediction in breast cancer

**DOI:** 10.1038/s41523-023-00577-4

**Published:** 2023-08-30

**Authors:** Sangjoon Choi, Soo Ick Cho, Wonkyung Jung, Taebum Lee, Su Jin Choi, Sanghoon Song, Gahee Park, Seonwook Park, Minuk Ma, Sérgio Pereira, Donggeun Yoo, Seunghwan Shin, Chan-Young Ock, Seokhwi Kim

**Affiliations:** 1 Department of Pathology and Translational Genomics, Samsung Medical Center, Sungkyunkwan University School of Medicine, Seoul, Republic of Korea; 2 Lunit Inc, Seoul, Republic of Korea; 3 Department of Pathology, Ajou University School of Medicine, Suwon, Republic of Korea; 4 Department of Biomedical Sciences, Ajou University Graduate School of Medicine, Suwon, Republic of Korea

**Keywords:** Breast cancer, Predictive markers

## Abstract

Tumor-infiltrating lymphocytes (TILs) have been recognized as key players in the tumor microenvironment of breast cancer, but substantial interobserver variability among pathologists has impeded its utility as a biomarker. We developed a deep learning (DL)-based TIL analyzer to evaluate stromal TILs (sTILs) in breast cancer. Three pathologists evaluated 402 whole slide images of breast cancer and interpreted the sTIL scores. A standalone performance of the DL model was evaluated in the 210 cases (52.2%) exhibiting sTIL score differences of less than 10 percentage points, yielding a concordance correlation coefficient of 0.755 (95% confidence interval [CI], 0.693–0.805) in comparison to the pathologists’ scores. For the 226 slides (56.2%) showing a 10 percentage points or greater variance between pathologists and the DL model, revisions were made. The number of discordant cases was reduced to 116 (28.9%) with the DL assistance (*p* < 0.001). The DL assistance also increased the concordance correlation coefficient of the sTIL score among every two pathologists. In triple-negative and human epidermal growth factor receptor 2 (HER2)-positive breast cancer patients who underwent the neoadjuvant chemotherapy, the DL-assisted revision notably accentuated higher sTIL scores in responders (26.8 ± 19.6 vs. 19.0 ± 16.4, *p* = 0.003). Furthermore, the DL-assistant revision disclosed the correlation of sTIL-high tumors (sTIL ≥ 50) with the chemotherapeutic response (odd ratio 1.28 [95% confidence interval, 1.01–1.63], *p* = 0.039). Through enhancing inter-pathologist concordance in sTIL interpretation and predicting neoadjuvant chemotherapy response, here we report the utility of the DL-based tool as a reference for sTIL scoring in breast cancer assessment.

## Introduction

Tumor-infiltrating lymphocytes (TILs) are mononuclear immune cells that penetrate into and around solid tumor areas^1^. TILs are observed in breast cancer tissue regardless of histologic and molecular subtypes, but triple-negative breast cancers (TNBC) and human epidermal growth factor 2 (HER2)-positive breast cancers tend to show more prominent TIL infiltration compared to hormone receptor-positive cancers^2^. The presence of TILs in breast cancer has been reported to be a prognostic factor and a predictor of response to chemotherapy and immune checkpoint inhibitors^3–7^.

Despite the importance of the TIL, previous studies have shown substantial interobserver variation among pathologists in determining TIL scores. To increase the objectivity of the TIL interpretation, the International Immuno-Oncology Working Group proposed a guideline that recommends the evaluation of only stromal TILs (sTILs) rather than the inclusion of intratumoral TILs (iTILs) and described standardized scoring methodology^8,9^. Nevertheless, the concordance rate of the TIL scoring has not been reported to be sufficiently high among pathologists^10–12^, which raises an urgent need for a novel method that can reduce the interobserver variation of sTIL scoring in breast cancer and lead to an accurate prediction of clinical outcomes.

Recently, deep learning (DL) has been increasingly applied in medical fields^13,14^. The adoption of digital pathology in the conventional pathologic workflow allows DL algorithms to be easily utilized in the analysis of digitalized histologic images^14–18^. The DL application in pathology is expected to reduce interobserver variability, as the International Immuno-Oncology Working Group suggested a computational assessment of TILs^19–21^. To be integrated into actual clinical practice, DL models require thorough validations^22^. However, clinical evidence for the role of the DL model to assist pathologists in evaluating TILs has not been sufficient to date. We previously developed and demonstrated a DL-based TIL analyzer and a Programmed death-ligand 1 analyzer that enabled not only an objective histologic evaluation, leading to the elimination of interobserver variability, but also better prediction of therapeutic responses^23,24^.

**Fig. 1:**
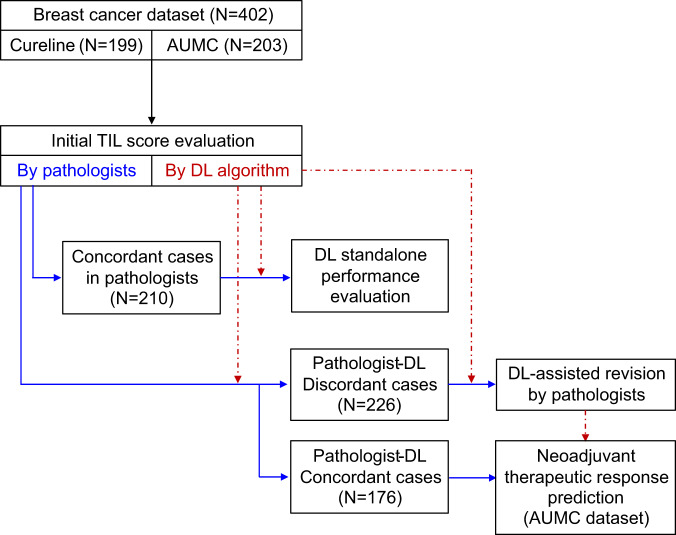
Overall workflow of data collection, evaluation of the standalone performance of deep learning (DL) model, DL-assisted pathologists’ revision, and the neoadjuvant chemotherapy response prediction. DL standalone performance was evaluated in cases that were concordant among pathologists (*N* = 210). In cases where there was disagreement between pathologists and DL (*N* = 226), pathologists performed the DL-assisted revision. Then the results were applied to neoadjuvant therapeutic response prediction.

In this study, we report the utility of a DL-based TIL analyzer in reducing the interobserver variation in sTIL scoring in breast cancers. Board-certified pathologists initially evaluated sTIL scores from whole slide images (WSIs), and concordant cases were utilized to evaluate the standalone performance of the DL model. For cases with a discrepancy between the pathologist and the DL model, each pathologist re-evaluated the cases with the assistance of a DL-based analyzer. The differences in sTIL level between responders and non-responders to neoadjuvant chemotherapy before and after DL assistance, respectively, were evaluated in TNBC and HER2-positive breast cancer (Fig. 1). With the aid of the DL-based TIL analyzer, the concordance rate of the sTIL scoring was significantly improved, and the prediction of therapeutic responses was enhanced. Our study demonstrates the value of DL assistance as a complementary tool for objective pathologic sTIL interpretation in breast cancer.

## Results

### Dataset characteristics

A dataset of WSIs (*N* = 402) was prepared for sTIL evaluation, consisting of 199 anonymized breast cancer histologic images obtained from Cureline (Brisbane, CA, USA) and 203 slides obtained from Ajou University Medical Center (AUMC) (Suwon, Republic of Korea). All specimens from Cureline were surgically resected primary breast cancer from treatment-naïve female patients. The specimens from AUMC were either HER2-positive breast cancer (*N* = 148) or TNBC (*N* = 55), which were core-biopsied before neoadjuvant chemotherapy between January 2014 and June 2022. The therapeutic response was evaluated in the surgically resected specimen following the neoadjuvant chemotherapy. Detailed clinicopathologic characteristics of the cases are summarized in Table 1.

**Table 1:** Clinicopathologic characteristics of 402 breast cancer patients in the external validation dataset.

Characteristics	All (*N* = 402)	Cureline (*N* = 199)	Ajou University Medical Center (*N* = 203)
Age [median, range]	56 [30, 87]	60 [30, 87]	53 [30, 73]
*Ethnicity*			
White patients (%)	160 (39.8%)	160 (80.4%)	0 (0.0%)
Asian patients (%)	242 (60.2%)	39 (19.6%)	203 (100.0%)
*Molecular subtype*			
Human epidermal growth factor receptor 2 (HER2)-positive	169 (42.0%)	21 (10.6%)	148 (72.9%)
Triple negative breast cancer (TNBC)	70 (17.4%)	15 (7.5%)	55 (27.1%)
Others or not available (N/A)	163 (40.6%)	163 (81.9%)	0 (0.0%)
*Histologic subtype*			
Invasive ductal carcinoma (IDC)	350 (87.1%)	163 (81.9%)	187 (92.1%)
Invasive lobular carcinoma (ILC)	21 (5.2%)	14 (7.0%)	7 (3.4%)
Mixed IDC and ILC	18 (4.5%)	17 (8.5%)	1 (0.5%)
Others	13 (3.2%)	5 (2.5%)	8 (3.9%)
*Bloom–Richardson grade*			
Grade I (well differentiated)	35 (8.7%)	23 (11.6%)	12 (5.9%)
Grade II (moderately differentiated)	206 (51.2%)	102 (51.3%)	104 (51.2%)
Grade III (poorly differentiated)	159 (39.6%)	74 (37.2%)	85 (41.9%)
N/A	2 (0.5%)	0 (0.0%)	2 (1.0%)
*Black’s nuclear grade*			
Grade 1	8 (2.0%)	0 (0.0%)	8 (3.9%)
Grade 2	189 (47.0%)	126 (63.3%)	63 (31.0%)
Grade 3	205 (51.0%)	73 (36.7%)	132 (65.0%)
*Cancer stage*			
I	34 (8.5%)	31 (15.6%)	3 (1.5%)
II	266 (66.2%)	123 (61.8%)	143 (70.4%)
III	88 (21.9%)	32 (16.1%)	56 (27.6%)
N/A	14 (3.5%)	13 (6.5%)	1 (0.5%)

### Initial TIL scoring by pathologists for concordance evaluation

Four pathologists participated in the assessment of the dataset (S. Choi [A], W. Jung [B], S. Kim [C], and T. Lee [D]) to classify a concordant set for the evaluation of the standalone performance of DL (Fig. 1). Each slide in the entire dataset was evaluated by three pathologists. Specifically, pathologists A and C reviewed all cases, and pathologists B and D evaluated 256 (all Cureline cases and 57 AUMC cases) and 146 cases (AUMC cases), respectively. The average sTIL score (%) of three pathologists for the whole dataset was 22.7 ± 19.3 (mean ± standard deviation [SD]). The average sTIL scores of each pathologist were 16.5 ± 16.6 (A), 27.7 ± 23.0 (B), 25.8 ± 24.1 (C), and 22.3 ± 17.5 (D), respectively. Of 402 cases, greater than a 10-percentage point difference in the sTIL score between at least two pathologists was observed in 192 cases (47.8%), and these were allocated in the discordant set. A total of 210 cases (52.2%) showed less than a 10-percentage point difference, which was included in the concordant set and utilized for evaluation of the standalone performance of the DL model.

### Standalone performance validation of the DL model

**Fig. 2:**
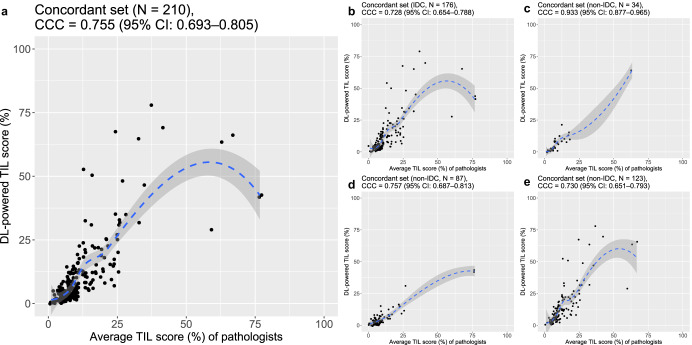
Evaluation of the standalone performance of the deep learning (DL)-based TIL analyzer. **a** The concordance correlation coefficient (CCC) in the initially concordant dataset (*N* = 210) between average stromal tumor-infiltrating lymphocyte (sTIL) score among the pathologists and the DL model interpretation. **b**, **c** The CCC values between average sTIL score among the pathologists and the DL model interpretation **b** in invasive ductal carcinoma cases (IDC) (*N* = 176) and **c** in other histologic subtype cases (non-IDC) (*N* = 34). **d**, **e** The CCC values between average sTIL score among the pathologists and the DL model interpretation **d** in the Cureline dataset (*N* = 87) and **e** in the Ajou University Medical Center (AUMC) dataset (*N* = 123).

A standalone performance of the DL-based TIL analyzer was evaluated in the concordant set (*N* = 210). The concordance correlation coefficient (CCC) value between the average sTIL score among three pathologists and the DL calculated score was 0.755 (95% CI: 0.693–0.805) (Fig. 2a). The performance of the DL model was acceptable regardless of histologic subtype or histologic grade of tumors. The CCC value for assessment of non-invasive ductal carcinoma (non-IDC) (*N* = 34, 0.933 [95% CI: 0.877–0.965]) was higher than that of IDC (*N* = 176, 0.728 [95% CI: 0.654–0.788]) (Fig. 2b, c). By histologic grade, the CCC value of Bloom–Richardson (B–R) grade II tumors was the highest (*N* = 117, 0.767 [95% CI: 0.688–0.828]), followed by grade III (*N* = 66, 0.711 [95% CI: 0.574–0.810]) and grade I tumors (*N* = 26, 0.626 [95% CI: 0.382–0.788]) (Supplementary Fig. 1a–c). Data from AUMC and Cureline showed similar CCC values (*N* = 123, 0.730 [95% CI: 0.651–0.793], and *N* = 87, 0.757 [95% CI: 0.687–0.813], respectively) (Fig. 2d, e). When evaluated in the larger set, including not only initially concordant cases but also the concordant cases after the DL-assisted revision (*N* = 286), a higher CCC value, 0.911 (95% CI: 0.889–0.928) was noticed (Supplementary Fig. 2). Supplementary Fig. 3 showed representative images with pathologists’ consensed sTIL score and DL-interpreted sTIL score.

### Initial concordance of TIL score among pathologists and comparison to DL interpretation

**Fig. 3:**
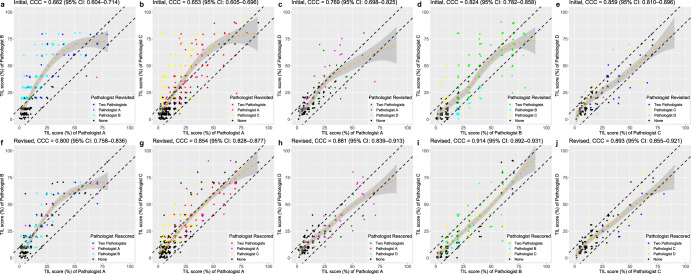
Deep learning (DL)-assisted reduction of interobserver variation in the stromal tumor-infiltrating lymphocyte (sTIL) score between pathologists. The correlation of the sTIL score between pathologists **a**–**e** before and **f**–**j** after DL-based TIL analyzer assistance. The degree of correlation is measured by the concordance correlation coefficient (CCC). The term “revisited” indicates the case that is returned to pathologists for review with DL assistance, whereas “rescored” refers to the case that is actually revised after DL assistance.

**Fig. 4:**
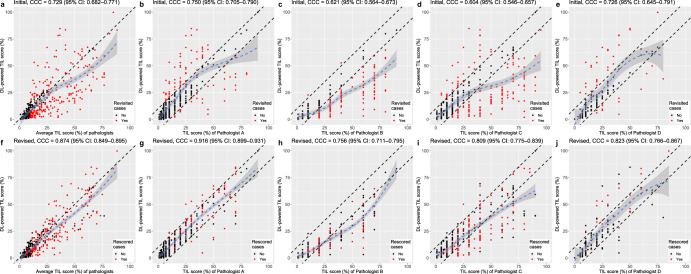
The correlation between the stromal tumor-infiltrating lymphocyte (sTIL) scores calculated by the deep learning (DL) model and the scores provided by pathologists. **a** The concordance correlation coefficient (CCC) between the value of the DL model and the average value of pathologists before DL-assisted revision. **b**–**e** The CCC between the value of the DL model and the value from each pathologist before DL-assisted revision. **f** The CCC between the value of the DL model and the average value of pathologists after DL-assisted revision. **g**–**j** The CCC between the value of the DL model and the value from each pathologist after DL-assisted revision.

The overall correlations of the initial sTIL score between two pathologists based on the CCC were 0.662 (95% CI: 0.604–0.714) for pathologists A and B, 0.653 (95% CI: 0.605–0.696) for pathologists A and C, 0.769 (95% CI: 0.698–0.825) for pathologists A and D, 0.824 (95% CI: 0.782–0.859) for pathologists B and C, and 0.859 (95% CI: 0.810–0.896) for Pathologists C and D (Fig. 3a–e), which were comparable with scores in previous reports (Supplementary Table 1)^10–12,25^. The coefficient of variation (COV) of the initial sTIL evaluation by three pathologists was 0.412 ± 0.254. The CCC values between the sTIL scores calculated by pathologists and those calculated by the DL model were 0.729 (for the average of three pathologists, 95% CI: 0.682–0.771), 0.750 (for pathologist A, 95% CI: 0.705–0.790), 0.621 (for pathologist B, 95% CI: 0.564–0.673), 0.604 (for pathologist C, 95% CI: 0.546–0.657), and 0.726 (for pathologist D, 95% CI: 0.645–0.791) in the whole dataset (Fig. 4a–e). In 167 (41.5%) cases, the sTIL scores calculated by the DL model were within the range of scores given by the pathologists. The scores by the DL model were lower than the range by the pathologists in 174 (43.3%) cases. Sixty-one (15.2%) cases had scored by the DL model that exceeded the range of scores by the pathologists.

### DL-assisted revision of the TIL score by pathologists

**Fig. 5:**
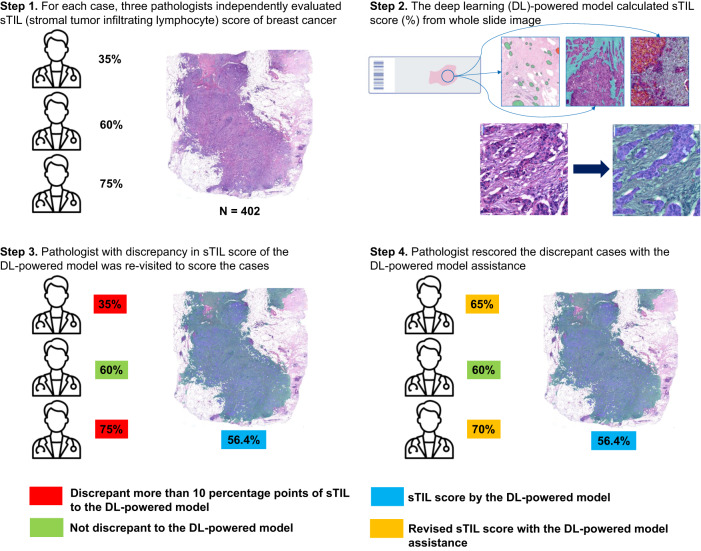
The detailed workflow of deep-learning (DL)-assisted revision of stromal tumor-infiltrating lymphocyte (sTIL) by pathologists. Pathologists and DL independently performed the sTIL assessment (Step 1 and 2). If there was more than a 10% difference between each pathologist and DL results (Step 3), the pathologist performed a reassessment with the DL-powered model assistance (Step 4).

According to the 10-percentage point difference cutoff from the DL-interpreted value, 92 (22.9%, Pathologist A), 130 (50.8%, Pathologist B), 157 (39.1%, Pathologist C), and 40 (27.4%, Pathologist D) slides were subjected to re-evaluation by each pathologist (Fig. 5). In all, 226 (56.2%) slides were reviewed by at least one pathologist (49 slides by three pathologists, 95 slides by the two pathologists, and 82 slides by one pathologist) (Fig. 1). The re-examined slides showed lower CCC values among three pathologists than cases that were not re-examined (Supplementary Table 2). Among those slides, the pathologists changed their initial sTIL score in 86 (93.5%, Pathologist A), 91 (70.0%, Pathologist B), 130 (82.8%, Pathologist C), and 24 (60.0%, Pathologist D) cases.

**Fig. 6:**
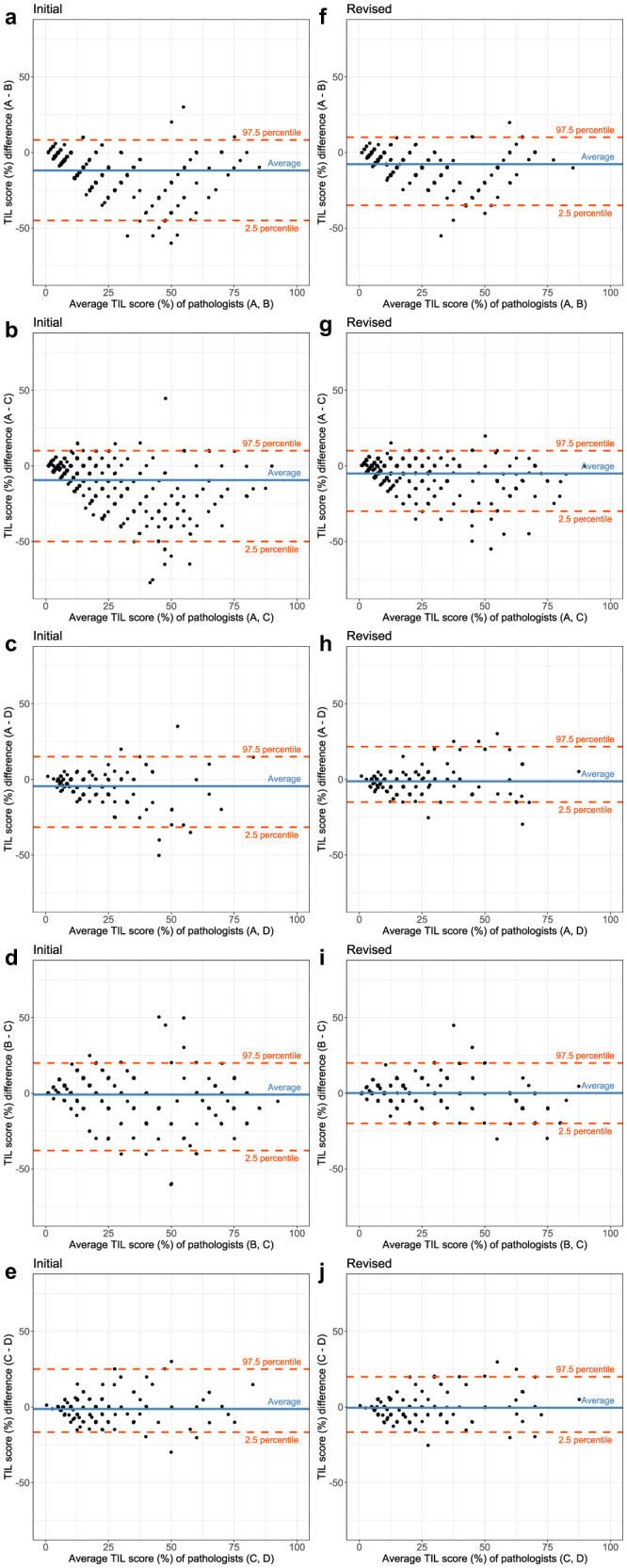
Bland–Altman plots displaying the changes of interobserver variation on stromal tumor-infiltrating lymphocyte (TIL) scoring. Before (**a**–**e**) and after deep learning assistance (**f**–**j**). Straight blue lines are the average difference between the two pathologists and dashed red lines indicate the 95% confidence interval.

In the discrepant cases, the number of slides showing more than a 10-percentage point difference in the sTIL score between at least two pathologists was decreased to 116 cases (28.9%; *p* < 0.001; McNemar test) after DL-assisted revision. The CCC values between the two pathologists also consistently increased (Fig. 3f–j). The CCC values between the pathologists and the DL model were increased to 0.874 (for all three pathologists, 95% CI: 0.849–0.895), 0.916 (Pathologist A, 95% CI: 0.899–0.931), 0.756 (Pathologist B, 95% CI: 0.711–0.795), 0.809 (Pathologist C, 95% CI: 0.775–0.839), and 0.823 (Pathologist D, 95% CI: 0.766–0.867) in the whole dataset (Fig. 4f–j). The increases in CCC values were observed in both the Cureline set (Supplementary Fig. 4a, b) and the AUMC set (Supplementary Fig. 4c, d). The effect of DL-assisted revision was profound in the initially discordant set (*N* = 192, Supplementary Fig. 5). The COV of the revised sTIL score was 0.325 ± 0.225, which was significantly lower than the initial value (*p* < 0.001; paired *t*-test). The interobserver variances of sTIL scoring between two pathologists before and after DL assistance are depicted by Bland–Altman plots in Fig. 6. The initial differences in mean sTIL score between two pathologists were 13.0 ± 12.6 (Pathologist A and B), 11.6 ± 13.9 (Pathologist A and C), 7.3 ± 8.7 (Pathologist A and D), 9.9 ± 10.9 (Pathologist B and C), and 6.8 ± 6.6 (Pathologist C and D), and consistently improved to 8.8 ± 9.0 (*p* < 0.001; paired *t*-test), 6.9 ± 8.6 (*p* < 0.001), 6.2 ± 6.7 (*p* < 0.001), 5.7 ± 7.0 (*p* = 0.115), and 6.1 ± 6.4 (*p* = 0.238) after DL assistance.

### Potential biases by pathologists and DL model in evaluating TIL

**Fig. 7:**
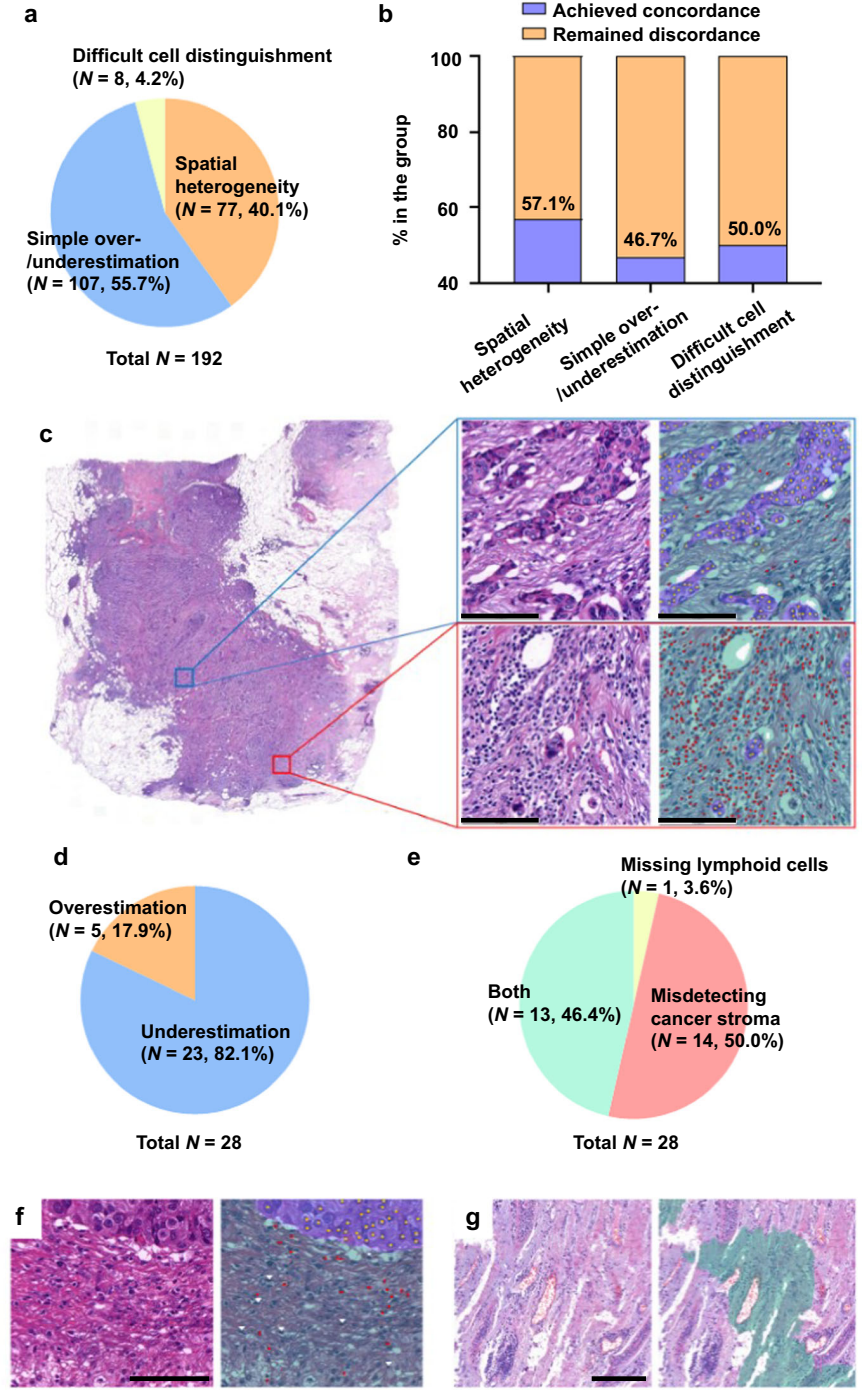
Biases of tumor-infiltrating lymphocyte (TIL) evaluation by pathologists and deep learning (DL)-powered TIL analyzer. **a** Classification of the initial discordance cases of TIL interpretation by pathologists. **b** Degree of achieving concordance by the DL assistance according to the initial discordance categories. **c** Example of an uneven stromal TIL (sTIL) distribution. The area in the blue box represents a low sTIL area, while the area in the red box represents a high sTIL area (scale bar, 250 μm). **d** Classification of the estimation error by DL-powered TIL analyzer. **e** Classification of the DL misinterpretations by causes. **f** Example of missed lymphoid cells by the DL-powered TIL analyzer (scale bar, 100 μm). **g** Example of incorrect cancer stroma segmentation by the DL-powered TIL analyzer (scale bar, 250 μm). Red dot, lymphocytes detected by DL model; white arrowhead, lymphoid cells missed by DL model; yellow dot: tumor cells detected by DL model; green region, cancer stroma segmented by DL model; purple region, cancer area segmented by DL model.

After the evaluation by the pathologists, the interaction between the pathologist and the DL model was analyzed post hoc. First, we reviewed the WSIs of 192 cases in the discordant set, showing the sTIL score difference greater than 10 percentage points between pathologists. More than half of the discrepancy stems from a simple over-/underestimation of lymphoid cells (*N* = 107, 55.7%). Also, the spatially heterogeneous distribution of the lymphoid cells turned out to be a significant cause of the initial discordance (*N* = 77, 40.1%). In some TNBC cases, the small-sized tumor cells were difficult to distinguish from lymphoid cells, especially in the slides with squeezing or cauterization artifacts (*N* = 8, 4.2%) (Fig. 7a). The DL-assisted revision could decrease the initial sTIL score differences to less than 10-percentage points, especially in the samples showing the heterogeneous distribution of lymphoid cells (Fig. 7b, c). The cases where the initial discrepancy was resolved by DL-assisted revision had significantly higher COVs than the other slides (0.560 ± 0.255 vs. 0.384 ± 0.245; *p* = 0.001; student *t*-test).

Next, we analyzed 28 cases in which DL model misinterpretation was suspected. These cases included those for which (1) there was a difference of 10 percentage points or more in the sTIL score between the DL model and all pathologists after re-evaluation, (2) two or more pathologists revisited but did not change the initial sTIL score, and (3) pathologists revised the score in the opposite direction to the value of the DL model (for example, a pathologist gave the initial sTIL score as 30% and the DL model suggested 10%, but the pathologist revised the sTIL score to be 40%). A thorough review of these cases revealed that the sTIL score was underestimated in the majority of the cases (Fig. 7d). The DL model misidentified cancer stroma in 14 cases (50.0%), missed lymphoid cells in one case (3.6%) and misdetected both entities in 13 cases (46.4%), as shown in Fig. 7e–g.

### Improved prediction of neoadjuvant chemotherapy response by DL-assisted TIL scoring

**Fig. 8:**
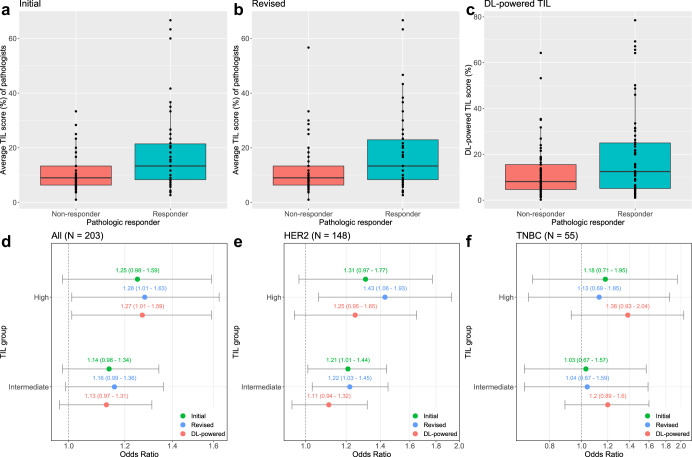
Enhanced prediction of neoadjuvant chemotherapy response with deep learning (DL)-assisted stromal tumor-infiltrating lymphocyte (sTIL) evaluation. **a**, **b** The average sTIL score in the responder group (Miller–Payne grade 4 and 5) and the non-responder group (Miller–Payne grade 1–3) was interpreted by pathologists without (**a**) and with DL-assistance (**b**). **c** The DL standalone interpretation of sTIL score in the responder and the non-responder group (The center lines in the boxplot represent median values; the bounds of the boxplot represent the interquartile ranges; the whiskers represent the range of the data). **d**–**f** The odds ratio (OR) of sTIL high group (sTIL ≥ 50) and intermediate group (sTIL 1–49) compared to sTIL low group (sTIL < 10) in relation to the neoadjuvant chemotherapeutic response, **d** in the entire HER2-positive breast cancer and triple-negative breast cancer (TNBC) dataset, **e** in the HER2-positive breast cancers, and **f** in the TNBCs (error bars, 95% confidence interval). * denotes *p* < 0.05, and ** denotes *p* < 0.01. The *p*-values were calculated using a student’s *t*-test for (**a**–**c**) and binary logistic regression for (**d**–**f**).

It is well-documented that high sTIL distribution in HER2-positive and TNBC is a predictor of better response to neoadjuvant chemotherapy^26–32^. Consistently, responders (*N* = 92) to the neoadjuvant chemotherapy (Miller-Payne grade 4 and 5; reduction of more than 90% of tumor cells) had higher initial sTIL score evaluated by three pathologists compared to the non-responder (*N* = 111, Miller-Payne grade 1–3) (mean ± standard deviation: 25.4 ± 18.8 vs. 19.0 ± 15.4; *p* = 0.010; student *t*-test) (Fig. 8a). The DL-assisted revision could increase the difference of average sTIL score between responders and non-responders (26.8 ± 19.6 vs. 19.0 ± 16.4; *p* = 0.003) (Fig. 8b). The standalone interpretation by DL model could also reveal the difference of sTIL score between the responder and non-responder group at a similar level to the revised interpretation by pathologists (27.1 ± 22.2 vs. 18.6 ± 18.3; *p* = 0.004) (Fig. 8c).

Also, binary logistic regression analysis revealed that the sTIL-high group (sTIL ≥ 50) was related to the responder compared to the sTIL-low group (sTIL < 10) as shown in odds ratios (ORs) in Fig. 8d. The correlation was insignificant with the initial sTIL scoring by pathologists, however, the revised score with DL-assistance and the DL standalone interpretation data showed statistical significance (OR 1.25 [95% CI: 0.98–1.59]; *p* = 0.076 for initial pathologists’ reading; OR 1.28 [95% CI: 1.01–1.63]; *p* = 0.039 for DL-assisted revised reading; OR 1.27 [95% CI: 1.01–1.59]; *p* = 0.039 for DL standalone interpretation; binary logistic regression). For the sTIL-intermediate group (sTIL 10–49) compared to the sTIL-low group, statistical significance was not reached in all three groups (OR 1.14 [95% CI: 0.98–1.34]; *p* = 0.095 for initial pathologists’ reading; OR 1.16 [95% CI: 0.99–1.36]; *p* = 0.060 for DL-assisted revised reading; OR 1.13 [95% CI: 0.97–1.31]; *p* = 0.114 for DL standalone interpretation) when analyzed in the entire AUMC dataset including both TNBC and HER2-positive tumors (total *N* = 203). However, in HER2-positive patients (*N* = 148), correlation to neoadjuvant chemotherapeutic response was identified in the sTIL-intermediate group compared to the sTIL-low group with initial and DL-assisted sTIL interpretation by pathologists (OR 1.21 [95% CI: 1.01–1.44]; *p* = 0.036 for initial pathologists’ reading; OR 1.22 [95% CI: 1.03–1.45]; *p* = 0.022 for DL-assisted revised reading; OR 1.11 [95% CI: 0.94–1.32]; *p* = 0.229 for DL standalone interpretation). The OR of the sTIL-high group in HER2-positive tumors was higher with the DL-assisted interpretation by pathologists in comparison to the result of the entire dataset (OR 1.43 [95% CI: 1.06–1.93]; *p* = 0.020). The ORs of the sTIL-high group in HER2-positive patients were statistically insignificant for initial pathologists’ interpretation or DL standalone interpretation (OR 1.31 [95% CI: 0.97–1.77]; *p* = 0.084 for initial pathologists’ reading; OR 1.25 [95% CI: 0.95–1.65]; *p* = 0.115 for DL standalone interpretation) (Fig. 8e). In TNBC patients (*N* = 55), neither the sTIL-high group nor the sTIL-intermediate group was correlated to the therapeutic response when compared to sTIL-low group, in initial pathologists’ interpretation (OR 1.18 [95% CI: 0.71–1.95]; *p* = 0.518 for sTIL-high group; OR 1.03 [95% CI: 0.67–1.57]; *p* = 0.907 for sTIL-intermediate group), DL-assisted revised interpretation (OR 1.13 [95% CI: 0.69–1.85]; *p* = 0.634 for sTIL-high group; OR 1.04 [95% CI: 0.67–1.59]; *p* = 0.873 for sTIL-intermediate group), and DL standalone interpretation (OR 1.38 [95% CI: 0.93–2.04]; *p* = 0.116 for sTIL-high group; OR 1.20 [95% CI: 0.89–1.60]; *p* = 0.235 for sTIL-intermediate group) (Fig. 8f). When the correlations of five individual Miller–Payne grade categories and sTIL scores were evaluated, the initial pathologists’ consensus, revised pathologists’ consensus, and the DL model consistently showed similarities (Supplementary Fig. 6).

## Discussion

Here we report that assistance with a DL-based TIL analyzer can lead to reduced interobserver variation among pathologists in evaluating sTILs in breast cancer compared with their interpretation without DL assistance. Despite the importance of TIL evaluation in breast cancer, only a few studies report that lymphocytes can be objectively identified using DL algorithms^33–35^. Also, whether DL assistance can help pathologists provide more accurate TIL scores has not yet been investigated.

Several meta-analyses reveal that TIL distribution is associated with disease prognosis and response to treatment^36–39^. High TIL levels are associated with increased pathological complete response rates to neoadjuvant chemotherapy in TNBC and HER2-positive breast cancer^36^. Additionally, accumulating evidence shows that high TIL content in breast cancers is associated with increased Programmed death-ligand 1 expression and better responses to immune checkpoint inhibitors^2,36,40^. Recent studies report that stratification of breast cancer patients according to the sTIL level has as much prognostic value as stratification by cancer stage^6,41^. Therefore, the sTIL level is now regarded as one of the most significant findings in the histopathologic examination of breast cancer and is recommended by international guidelines to be incorporated into the pathology report^42–44^.

However, the interobserver variation in sTIL evaluation among pathologists has long been problematic^12,45^. An objective calculation of sTILs is difficult due to its complexity; pathologists should classify lymphocytes from various cells, segment the stromal area, and then calculate the sTIL score. Although current guidelines provide standards of interpretation for pathologists, histomorphologic evaluation is inevitably subjective^25,46^. Supplementary Table 1 summarizes the interobserver concordance of breast cancer sTIL evaluation reported in previous studies^10–12,25^. The interobserver variation is especially prominent when general pathologists participate in the studies compared with when the examinations were conducted only by specialists in breast pathology.

The role of digital pathology in medicine has grown over time and has included larger storage for scanned WSIs and an increase in computational power^17,18^. DL algorithms are successfully introduced to analyze WSIs of solid tumors, including breast cancer^21,47^, and several studies apply DL algorithms to TIL evaluation in breast cancer^33–35,48–50^. Those studies report comparable TIL estimation by DL models and manual TIL evaluation by pathologists, although additional validation is required due to the relatively small number of datasets on which the models were based^33–35^. Considering that the pathologist’s interpretation of the TIL score, not the evaluation by a DL model, is still the gold standard in TIL analysis, studies on the process of evaluating TILs more accurately and objectively through the interaction between a pathologist and a DL model are needed. A recent report also recommends that a DL model for TILs needs to be a computer-aided tool for pathologists rather than a fully automated model that replaces human interpretation^20^.

In this study, we observed significant variability in sTIL assessments among pathologists, as previous studies have also reported^10–12,25^. The interobserver variability among pathologists was markedly reduced after DL-assisted revision. In particular, the concordance among the pathologists in this study—who are not specialists in breast pathology—was enhanced from the general pathologist level to the breast specialist level with DL assistance when referring to the correlation and agreement rates from the previous studies (Supplementary Table 1). This finding implies that our DL-based TIL analyzer can play an important role in correcting misinterpretations by pathologists. A common cause of interobserver variation in TIL evaluation is an unequal distribution of lymphocytes throughout the WSI because pathologists have difficulty estimating disproportionate cell counts in large fields^12^. Our DL model accurately determines the cases that exhibit an uneven distribution of TILs, as shown in Fig. 7c, implying that the DL model can practically solve one of the common difficulties in reading TILs at the WSI level.

In some cases, interobserver variation was still identified despite assistance from DL and the second chance to revise the interpretation. The inconsistency was mainly due to poor sample quality, such as squeezing artifacts and over/under-staining. The discrepant cases between the DL algorithm and the pathologists also frequently revealed a low quality of preparation. As shown in Fig. 7f, g, some lymphoid cells and areas of cancer stroma were missed by the DL model. Atypical histomorphology of cells and tissue resulting from preparation artifacts were frequently identified in these cases, which suggests insufficient training of the DL algorithms with suboptimal inputs. The performance degradation of the DL model is often observed when the staining, scanner, sample acquisition site, and demographics are different^18,51^, and when tissue artifacts are present in the training and/or internal validation sets^52–56^. Suboptimal quality images are unavoidable and are encountered in daily pathology practice; however, these errors may not lead to a poor decision if pathologists can directly verify the erroneous inference result of the DL model and reflect this finding in their final decision. From this point of view, DL model development in digital pathology should be directed toward the augmentation of decisions made by pathologists^18,57^.

It is thus noteworthy that the pathologists’ sTIL evaluation with DL assistance exhibited better prediction of neoadjuvant chemotherapy responses in patients compared to the evaluation without DL assistance, and the tumor response prediction with the DL standalone evaluation could lead to a comparable result. In our results, the average sTIL scores evaluated by pathologists or the DL model were higher in chemotherapy responders compared to non-responders in TNBCs and HER2-positive breast cancers, which is in line with the previous studies^26–32^. A more profound statistical significance was observed when the result of DL-assisted revision or that of DL standalone interpretation was utilized in the analysis, compared to that of initial pathologists’ interpretation. Also, the fact that the sTIL-high tumor group of initial pathologists’ interpretation had failed to show a significant correlation to the chemotherapy response, but statistical significance was observed in the group of DL-assisted revision highlights an ancillary role of DL in the accurate histologic interpretation. Imposingly, we could identify a similar level of correlation by the DL-based standalone reading. The DL-assisted improvement of therapeutic prediction was more evident in HER2-positive tumors. The DL-assisted change was insignificant TNBCs, possibly due to the relatively small number of datasets, because we had validated that our DL model could well-distinguish tumor cells and lymphoid cells not only in HER2-positive tumors but also in TNBCs. Further studies using a larger cohort consisting of multi-center patients with neoadjuvant chemotherapy information are imperative to demonstrate the efficacy of our DL-assisted sTIL evaluation.

Interestingly, we observed the better performance of the DL model on sTIL scoring in non-IDC, which are mostly composed of invasive lobular carcinoma (ILC), compared to IDC. Since ILC cells are small, round, monotonous, and discohesive than IDC cells, we first expected that the DL-based TIL analyzer could possibly misclassify the tumor cells as lymphocytes or plasma cells^20^. However, the superior performance of the DL model in ILC implies that the algorithm is successfully trained to distinguish the subtle morphological differences among the cell types. Considering that high sTIL infiltration is associated with unfavorable clinical outcomes in ILC^58^, a reliable sTIL evaluation by using our DL-based TIL analyzer can play a pivotal role in the enhanced prediction of the prognosis in this specific histologic subtype. Additionally, the standalone performance of the DL model was superior in tumors with histological grade II/III than in tumors with grade I. It is well-documented that TNBCs tend to display high histological grades than other molecular subtypes^59^. Considering the prognostic impact of sTILs in TNBC, the better performance of DL assessment in high histologic grade tumors indicates the usefulness of the DL model on sTIL evaluation and prediction of the prognosis of the TNBC.

The DL algorithm we developed and validated can help pathologists further understand the role of TILs in breast cancers and other types of cancer. For example, iTILs have important clinical value in breast cancer, but it is difficult to evaluate iTILs objectively on hematoxylin and eosin (H&E)-stained slides without additional special stains^60–63^. By applying a subtle modification, our DL model enables the detection and calculation of iTILs in addition to sTIL. Additionally, the distribution patterns of TILs and the TIL density, which can be accurately evaluated by DL algorithms, are recognized as important features in relation to molecular signatures^45,64–66^. DL-assisted TIL evaluation can explore and analyze unknown or complex patterns of TILs, which is fastidious, time-consuming, and associated with interobserver variation.

This study has some limitations. First, we analyzed 402 breast cancer slides that were evaluated by three pathologists. Although some previous studies investigate the concordance of breast sTIL evaluation between two-to-four pathologists^10,11,25,34,67^, it is obvious that a large number of participants is important for the accurate estimation of interobserver variation in the real-world setting^68^. Thus, to verify the performance of the model, it may be necessary to validate the model with a larger number of pathologists and larger, diverse dataset sources. Second, the DL model performance can be further improved because the sTIL evaluation results of the DL model and those of pathologists did not match well in a few cases, especially when pathologists interpreted high sTIL scores. In addition to improved cell detection and tissue segmentation performance of the DL model, it is necessary to advance the formula for calculating the sTIL score from cell detection and tissue segmentation results based on the verified ‘gold standard’ in breast cancer datasets. Third, for revisited cases, pathologists may have tended to rely on the DL model in the scoring of sTIL and changed their first assessment even if those were correct estimations. To reduce this error, pathologists should refer to the score of the DL model after careful examination of whether there is a considerable misinterpretation of the DL model on tissue segmentation or lymphocyte labeling. Additionally, comparing each pathologist’s sTIL scoring with and without DL assistance with an adequate washout period is required for more reliable results.

In conclusion, for the first time, we demonstrated that a DL-based TIL analyzer could help pathologists objectively and accurately evaluate sTILs in breast cancer by reducing interobserver variability. The DL-assisted sTIL evaluation allows better prediction for neoadjuvant chemotherapy response in patients with TNBCs and HER2-positive breast cancers. Further verification of the performance of this analyzer in routine clinical practice will reveal its potential value as a complementary and effective tool for objective pathologic interpretation.

## Methods

### Development of a DL-based TIL analyzer

A DL-based TIL analyzer, Lunit SCOPE IO (Lunit Inc., Seoul, Republic of Korea), was developed with data from 2.8 × 10^9^ μm^2^ H&E-stained tissue sections and 6.0 × 10^5^ TILs from 3166 WSIs of 25 cancer types annotated by board-certified pathologists, as previously described^23^. Among the dataset, 7.6 × 10^8^ μm^2^ H&E-stained tissue sections and 1.1 × 10^5^ TILs were from 557 WSIs of breast cancer (Supplementary Table 3). The pathologists segmented the cancer area and cancer stroma and annotated cancer cells and lymphoid cells from slide images to develop the DL model. All H&E-stained slides were prepared from formalin-fixed paraffin-embedded samples of surgically resected or biopsied tissue and were scanned at 40× magnification using a slide scanner (Pannoramic 1000, 3DHISTECH Ltd., Hungary).

We developed two complementary but separate DL models to evaluate the sTIL score: one for cell detection and the other for tissue segmentation, which was described in our previous study^23^. The cell detection model was applied to detect lymphoid cells in all areas, while the tissue segmentation model was applied to segment the stromal area in this study. Each model was trained independently, starting from an ImageNet-pre-trained backbone^69^. The models and training routines were implemented in the Python programming language (Version 3.7) and the PyTorch framework (Version 1.4)^70^.

The cell detection model detects the cancer cells and lymphoid cells on the WSIs and is based on the Faster R-CNN object detection algorithm^71^. Due to the large size, WSIs cannot be directly processed by the model. Therefore, tiles of size 1024 × 1024 pixels (0.04 mm^2^), extracted from WSIs, are used instead. The model was trained to predict the location of the cells of interest, a bounding box surrounding the nucleus of the cells, and the class of the cell. First, Faster R-CNN computes feature maps from a given tile through a backbone, which was defined as a ResNet-34 architecture^72^. Then, a Region Proposal Network (RPN) processes the features and predicts cell candidates. Finally, for each candidate, a Region of Interest (ROI) head regresses the position of the cell and bounding box. In contrast to the original Faster R-CNN algorithm, we employed the Dice loss function^73^ as the objective for cell classification. Additionally, when training the RPN network, we sampled positive (cell of interest) and negative (non-cell) cells with a 9:1 ratio to tackle the class imbalance in the task of cell detection. The cell detection model was trained by using the Adam optimizer^74^, with a learning rate of 0.001, and mini-batches of 32 samples. A variety of common data augmentation techniques were applied during training, including random shearing, random rotations, or random changes in image appearance (brightness, hue, contrast, and saturation).

The tissue segmentation model is based on a DeepLabv3 semantic segmentation model^75^, and a backbone is based on EfficientNet-B3^76^. The model classifies each pixel on the WSIs, whether it belongs to the cancer area, cancer stroma, or other background regions except the cancer area and cancer stroma. It takes input patches of size 448 × 448 pixels (0.11 mm^2^) and outputs prediction maps of size 112 × 112 units. The prediction maps are then linearly upsampled by a factor of 4 to match the original input patch size. Therefore, we obtain one prediction per input pixel. During training, the Dice loss function^73^ was optimized using Adam optimizer^74^ with a learning rate of 0.001. The learning rate was decayed by a factor of 0.1 after every 100 epochs. Similarly to the cell model, a variety of common data augmentation techniques were applied during training, including random shearing, random rotations, or random changes in image appearance (brightness, hue, contrast, and saturation).

The performance of the DL-based model on tumor cell and lymphocyte detection was 79.02% and 76.25% in F1-score, respectively, and on cancer area and cancer, stroma segmentation was 75.55% and 67.79% in intersection over union (IoU), respectively. In breast cancer, F1-score for tumor cell and lymphocyte detection was 85.61% and 78.87% in each, and IoU for cancer area and cancer stroma were 81.92% and 66.60%, respectively. To check the reproducibility of the model, we further randomly sampled with replacement (the same number of grids) from the available 1650 tissue and 1611 cell grids from 15 cancer types and conducted a bootstrapping evaluation repeatedly 100 times. In breast cancer, F1-score for tumor cell and lymphocyte detection was 85.67% and 77.95% in each, and IoU for cancer area and cancer stroma were 82.04% and 66.47%, respectively (Supplementary Fig. 7).

By combining the information, we could calculate the sTIL score in WSIs based on lymphoid cells only in the cancer stroma as in Eq. (1). Normalization is inevitable in the sTIL score equation because the DL model detects the actual area of the cancer stroma (area), but only identifies the presence of lymphoid cells and depict them as dots (not detecting the actual area occupied by the cells). Considering that lymphoid cells in breast cancer are regular in size (a sphere with a radius of 3 μm), a constant α can be defined and multiplied by the numerator to mathematically define the sTIL score. The constant α is determined by the following patch-level analysis.

1 $${\rm{stromal}}\,{\rm{tumor}}-{\rm{infiltrating}}\,{\rm{lymphocytes}}\,{\rm{score}}=\frac{\alpha \,X\,{\rm{lymphoid}}\,{\rm{cell}}\,{\rm{count}}}{{\rm{cancer}}\,{\rm{stroma}}\,{\rm{area}}}$$

For the development and validation of the sTIL score calculation algorithm, a total of 249 grids of 1024 × 1024 pixels were randomly cropped from breast cancer WSIs in The Cancer Genome Atlas (TCGA) dataset^77^. Next, the sTIL score was evaluated within the grid area by three pathologists (S. Choi, W. Jung, and S. Kim), and 171 of 249 grids were scored for sTIL by all three pathologists. The remaining 78 grids were excluded and were not evaluated by all three pathologists due to issues with the sample quality raised by at least one pathologist. Empirically, we set the α as 6.5, 7.0, and 7.5 for the algorithm and compared the correlation to the mean TIL score of pathologists. The α value 6.5 generally showed a good correlation value to the mean TIL score of pathologists, but it had a lower correlation value than 7.0 or 7.5 for sTIL scores less than 15 or 10 in most cases (Supplementary Table 4, Supplementary Fig. 8). On the other hand, multiplication of 7.5 showed a relatively good correlation in the sTIL score of less than 20, but the overall correlation in the entire sTIL range was inferior. Multiplication by 7.0 revealed a balanced result between the value of 6.5 and 7.5. Therefore, we utilized 7.0 as a constant α in the sTIL scoring equation. For the 171 grids, the correlation measured by CCC between the DL-calculated sTIL score and the average value obtained by the three pathologists was 0.776 (95% CI: 0.719–0.823). The ICC values between the sTIL scores of the DL model and those of each pathologist were 0.651 (95% CI: 0.585–0.709), 0.811 (95% CI: 0.765–0.849), and 0.710 (95% CI: 0.629–0.776), respectively.

Additionally, to further validate the selected *α* value as 7.0, we randomly selected 50 breast cancer WSIs from TCGA and had them evaluated by three pathologists, after which their results were compared with the DL model’s output. The pathologists evaluated sTIL scores for 48 among the 50 cases, except for the two cases with poor tissue or scan quality. The CCC between the DL-calculated sTIL score and the average sTIL score of the three pathologists’ interpretation was 0.874 (95% CI: 0.799–0.922) (Supplementary Fig. 9).

### Dataset

The Cureline dataset (*N* = 199) was obtained in WSIs of H&E slides with 40× magnification by the slide scanner mentioned above. The H&E slides of the AUMC dataset (*N* = 203) were scanned in WSIs by a Aperio AT2 digital whole slide scanner (Leica Biosystems Imaging, Buffalo Grove, IL, USA) at 40× magnification. The entire dataset was anonymized not to reveal the patients’ identification. The ethnic identity was determined by the researchers. The data were collected and utilized under the permission of the Institutional Review Boards (IRBs). For the Cureline dataset, the IRB from Saint Petersburg City Clinical Oncology Hospital approved the study under the protocol CU-2010 Oncology 12152009, and the IRB from Saint Petersburg Academic University of the Russian Academy of Sciences approved protocol number CU-M-07092015-C-INT. For the AUMC dataset, the IRB from Ajou University Medical Center approved the study under the protocol AJOUIRB-KS-2022-340. Informed consent was waived by the IRB because of the retrospective nature of the study and the anonymized clinical data used in the analysis. The study was performed in accordance with the Declaration of Helsinki.

### Initial evaluation of the TIL by pathologists

Four board-certified, non-breast pathologists evaluated the sTIL score at the whole slide level in the dataset by using a computer-based WSI viewer. The sTIL score was assessed based on the recommendations suggested by the International Immuno-Oncology working group^8^. Briefly, mononuclear lymphoid cells (lymphocytes and plasma cells) were counted as TILs, and polymorphonuclear leukocytes (neutrophils) were excluded. TILs in tumor areas with crush artifacts, necrosis, regressive hyalinization, and in the previous core biopsy site were excluded. Only TILs within the invasive tumor borders were scored, whereas TILs outside the tumor border and around ductal carcinoma in situ or normal lobules were excluded.

### Evaluation of the standalone performance of DL-based TIL analyzer

Following the initial assessment of sTIL in the entire dataset (*N* = 402) by pathologists, 210 cases (52.2%) showing less than a 10-percentage point difference among the pathologists’ interpretation was utilized to evaluate the standalone performance of the DL-based TIL analyzer. The CCC value was calculated between the average sTIL score among the pathologists and the DL interpretation. The same analyses were performed in the subgroups according to the datasets (Cureline or AUMC), histologic subtypes (IDC or non-IDC), and B-R histologic grade (grade I, II, or III) of tumors. The standalone performance of the DL model was once again assessed in the dataset including both initially concordant cases and the concordant cases following the DL-assisted revision.

### DL-assisted revision of the TIL score by pathologists

When the difference between the pathologist’s sTIL score and the DL model’s sTIL score exceeded a cutoff value of 10 percentage points, the cases were returned to the pathologists for re-evaluation (Figs. 1 and 5). These returned cases were defined as “revisited” cases. The pathologists reviewed the selected cases with software that provided DL assistance. The software displayed DL cell detection outputs (cancer cells and lymphoid cells) and area segmentation outputs (cancer area and cancer stroma) overlaid on the WSI (Fig. 7). When the pathologists revised their sTIL score of revisited cases after DL assistance, they were defined as “revised” cases. The correlation of the sTIL score between the DL model and the pathologist, as well as those among the pathologists, were evaluated.

### Effect of DL assistance on neoadjuvant chemotherapy response prediction

The pathological responses to neoadjuvant chemotherapy were assessed in 203 patients of the AUMC dataset by using the Miller-Payne grading system, which compares the cancer cellularity between the core needle biopsy sample and surgical resection specimen. The grading system is divided into five grades and as follows: grade 1, no reduction in overall cellularity; grade 2, a minor loss of tumor cells but overall high cellularity (up to 30% reduction); grade 3, between an estimated 30% and 90% reduction in cellularity; grade 4, a marked disappearance of more than 90% of tumor cells; grade 5, no invasive malignant cells in sections from the site of the tumor^78^. Grades 1–3 patients were classified as non-responders, and Grades 4 and 5 patients were classified as responders^26–28,79,80^. In addition, sTIL was divided into three groups, high sTIL (sTIL ≥ 50), intermediate sTIL (sTIL 10–49), and low sTIL (sTIL <10) and the binary logistic analysis was performed to figure out whether there was a difference in responders to chemotherapy according to the sTIL group. The difference in mean sTIL values obtained from the DL model and pathologists’ interpretation between responder and non-responder was also analyzed.

### Statistical analysis

Lin’s CCC was applied to evaluate the correlation of the sTIL score among the pathologists or between the DL model and each pathologist^81^. Bland-Altman plots were used to illustrate the changes in interobserver agreements on sTIL scores between pathologists before and after DL assistance^82^. Since the distribution of the sTIL evaluation results in this study follows a nonparametric, limit of agreement lines is indicated by 2.5/97.5 percentile. Differences in means or medians for a continuous variable between two groups were assessed by Student’s *t*-test or paired *t*-test. Categorical variables were compared using the chi-squared test or McNemar test. The COV was calculated to measure the dispersion of the distribution in sTIL evaluation by pathologists^83^. The result of binary logistic regression was shown as OR with 95% CI. All statistical analyses were performed using Python 3.7 and R version 4.0.3 software (R Foundation for Statistical Computing, Vienna, Austria).

### Reporting summary

Further information on research design is available in the Nature Research Reporting Summary linked to this article.

### Supplementary information

Supplementary material

Reporting summary

## Data Availability

The pathologists’ evaluation results and the inference results of the DL-powered model on 171 grids and 48 WSIs from TCGA can be accessed from the following link: https://figshare.com/articles/figure/Supplementary_Material_Inference_zip/23932503. The visualization and validation of the algorithm are available from the DL-powered model page, which is available upon request. The other data (Cureline and AUMC datasets) in this study are also available from the corresponding author for academic purpose-request.

## Code availability

The software was developed using Python programming language (version 3.7). The models are implemented using PyTorch v1.4 (available at https://github.com/pytorch/pytorch). The cell detection model is based on a proprietary implementation of Faster R-CNN (open-source implementations available online, e.g., at https://github.com/pytorch/vision/blob/main/torchvision/models/detection/faster_rcnn.py), with a ResNet-34 backbone architecture pre-trained on ImageNet data (pre-trained weights available at https://download.pytorch.org/models/resnet34-333f7ec4.pth and open-source implementation at https://github.com/pytorch/vision/blob/master/torchvision/models/resnet.py). The tissue segmentation model is based on a proprietary implementation of DeepLabV3 (open source implementations available online, e.g., at https://github.com/VainF/DeepLabV3Plus-Pytorch), with an EfficientNet-B3 backbone architecture pre-trained on ImageNet data (pre-trained weights available at http://storage.googleapis.com/public-models/efficientnet-b3-c8376fa2.pth and open-source implementation at https://github.com/lukemelas/EfficientNet-PyTorch). The data augmentation transformations and image manipulation routines are implemented using TorchVision v0.5.0 (https://github.com/pytorch/vision), Albumentations v0.4.5 (https://github.com/albumentations-team/albumentations), OpenCV Python v4.1.2.30 (https://github.com/opencv/opencv-python), and Scikit-image v0.16.2 (https://github.com/scikit-image/scikit-image). Mathematical and statistical operations are implemented using Numpy v1.18.1 (https://github.com/numpy/numpy), Pandas v1.0.1 (https://github.com/pandas-dev/pandas), Scipy v1.3.0 (https://github.com/scipy/scipy), and Scikit-learn v0.22.1 (https://github.com/scikit-learn/scikit-learn). Finally, the WSIs were read and manipulated using OpenSlide v3.4.1 (https://github.com/openslide/openslide) and corresponding Pyhton wrapper v1.1.1 (https://github.com/openslide/openslide-python). The R code and csv file to create the figures can be found at https://github.com/SooIckCho/Breast_TIL. For any questions regarding the replication of results, the corresponding author can be contacted.
