# Supplementary material for: Deep learning model improves tumor-infiltrating lymphocyte evaluation and therapeutic response prediction in breast cancer
Source: NPJ Breast Cancer. 2023 Aug 30;9:71. doi: 10.1038/s41523-023-00577-4 (PMC10469174; doi:10.1038/s41523-023-00577-4)
Supplement: Supplementary file 1 — Supplementary material [file 41523_2023_577_MOESM1_ESM.pdf]

Supplementary Table 1. Summary of interobserver concordance of breast tumor infiltrating lymphocyte (TIL) score in previous studies and this study. The superscript 'a' represents 'Concordance correlation coefficient', 'b' represents 'Cohen's kappa', 'c' represents 'Intraclass correlation coefficient', and 'd' represents 'Squared weighted kappa'.

	Number of participated pathologists (specialty)	Number of cases	Correlation	Agreement
Buisseret et al.[11]	3 (Breast pathologist [Br], General pathologist [Gen])	124	0.69[a]	Not available (N/A)
Swisher et al.[10]	4 (Br)	75	N/A	0.57[b]
Khoury et al.[25]	2 (Br)	100	0.91−0.96[c]	0.53−0.71[d]
Kos et al.[12] (Ring 1)	32 (Br, Gen)	60	0.70[c]	N/A
Kos et al. [12] (Ring 2)	28 (Br, Gen)	60	0.89[c]	N/A
Kos et al. [12] (Ring 3)	6 (Br, Gen)	100	0.76[c]	N/A
This study (Initial)	4 (Gen)	402	0.653−0.859[a]	N/A
This study (Revised)	4 (Gen)	192	0.800−0.914[a]	N/A

Supplementary Table 2. Concordance correlation coefficient values (95% confidence interval) between two pathologists in re-examined slides

	Re-evaluated slides with deep learning model (*N* = 226)	Slides not re-evaluated (*N* = 176)
Pathologist A vs. B	0.555 (0.471−0.629)	0.759 (0.667−0.827)
Pathologist A vs. C	0.524 (0.448−0.593)	0.856 (0.811−0.891)
Pathologist A vs. D	0.640 (0.487−0.755)	0.814 (0.728−0.875)
Pathologist B vs. C	0.709 (0.625−0.776)	0.840 (0.773−0.888)
Pathologist C vs. D	0.779 (0.663−0.858)	0.810 (0.722−0.873)

Supplementary Table 3. Training and validation dataset to development deep learning-based tissue analyzer, Lunit SCOPE IO

Source	Primary organ	Training data			Validation data		
		Slide No.	Annotated tissue (mm^2)	Annotated cell (no.)	Slide No.	Annotated tissue (mm^2)	Annotated cell (no.)
Samsung Medical Center	Breast	486	649.2	104,447	71	111.7	5,595
	Lung	145	42.3	14,308	5	4.0	97
	Others	1,572	513	120,730	173	154.2	18,896
Prostate, Lung, Colorectal, and Ovarian Cancer Screening Trial	Lung	238	329.0	146,615	101	102.8	4,404
iSpecimen	Lung	199	650.2	126,214	36	23.6	942
National Lung Screening Trial	Lung	72	150.9	45,911	20	14.8	397
Cureline	Lung	-	-	-	34	38.0	3,176
	Others	-	-	-	14	13.6	5,383
Total		2,712	2,334.7	558,225	454	462.7	38,881

Supplementary Table 4. Correlation between mean tumor infiltrating lymphocyte (TIL) of pathologists and deep learning based TIL analyzer, according to the different constant α. The degree of correlation is measured by concordance correlation coefficient.

**TIL range (mean of pathologists)**	**Constant α**		
	**6.5**	**7.0**	**7.5**
All (*N* = 171)	0.789 (0.731−0.835)	0.776 (0.719−0.823)	0.759 (0.702−0.806)
≤20 (*N* = 146)	0.660 (0.573−0.733)	0.662 (0.577−0.733)	0.658 (0.575−0.726)
≤10 (*N* = 122)	0.371 (0.271−0.464)	0.403 (0.296−0.500)	0.432 (0.321−0.532)

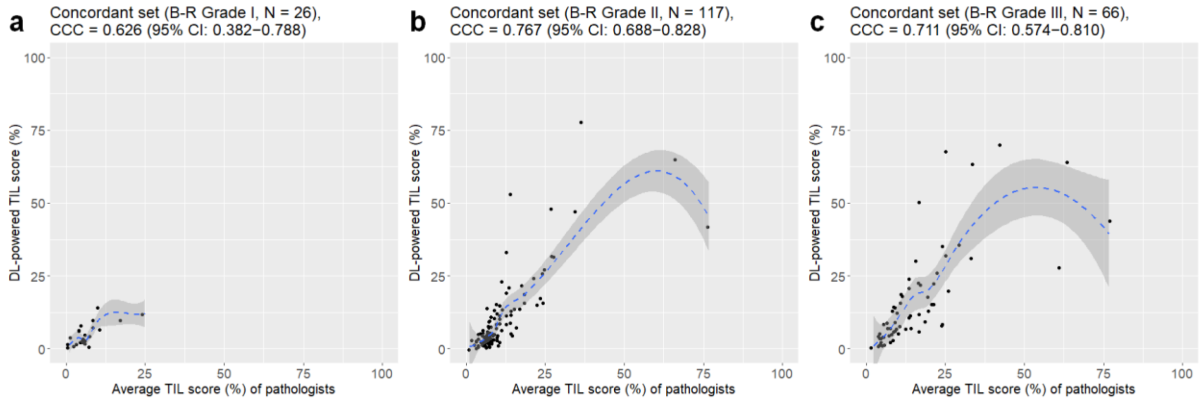

Supplementary Fig. 1. The concordance correlation coefficient (CCC) values between average stromal tumor infiltrating lymphocyte (sTIL) score among the pathologists and the deep learning (DL)-powered interpretation in Bloom-Richardson histologic grade I ($N = 26$) (**a**), II ($N = 117$) (**b**) and III ($N = 66$) (**c**) tumors.

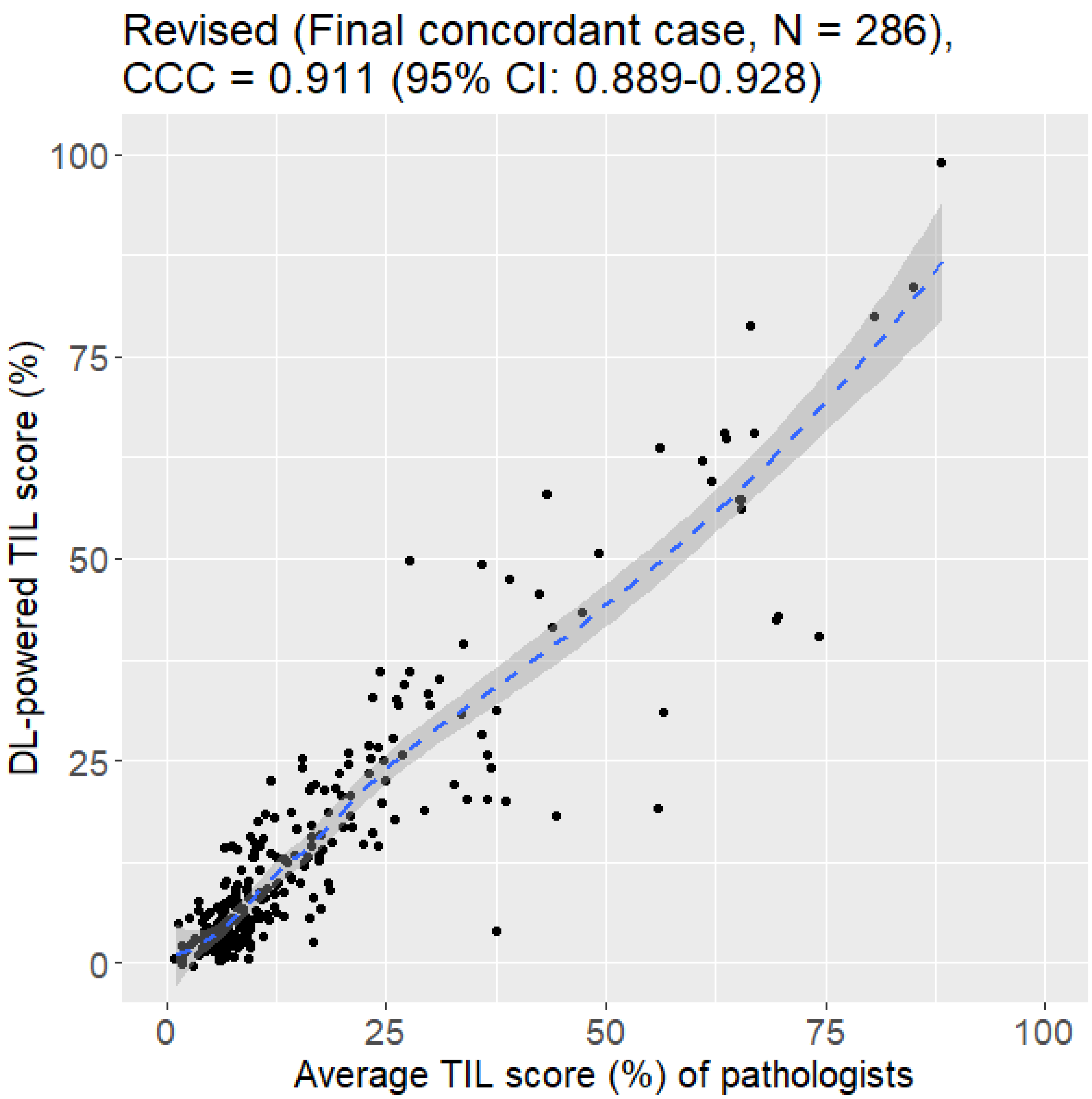

Supplementary Fig. 2. The standalone performance of the deep learning (DL) model in the dataset including the both initially concordant cases and the concordant cases following the DL -assisted revision, measured by concordance correlation coefficient (CCC).

250μm).

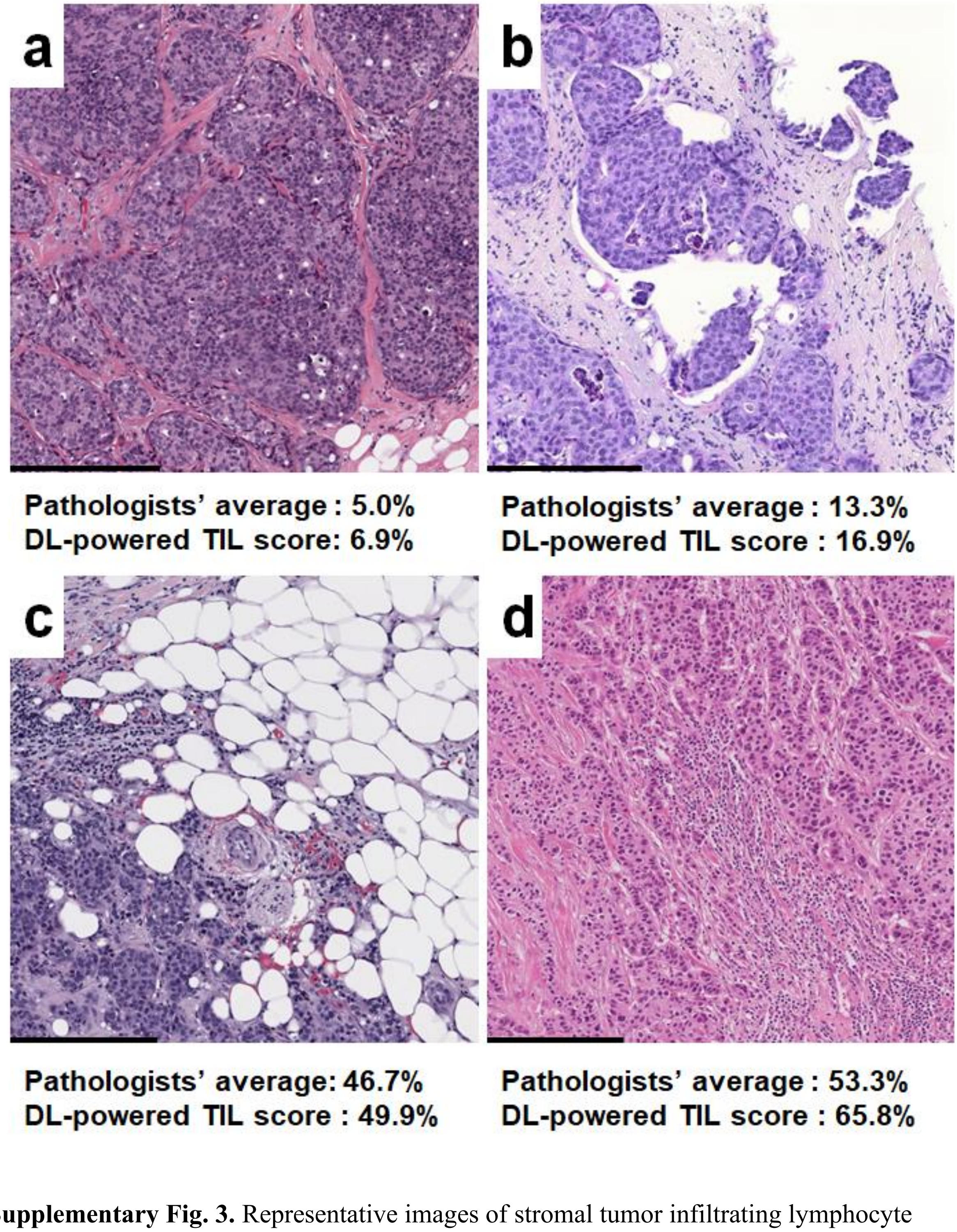

Supplementary Fig. 3. Representative images of stromal tumor infiltrating lymphocyte (sTIL) evaluation by pathologists and the deep learning (DL)-based TIL analyzer (scale bar, 250μm).

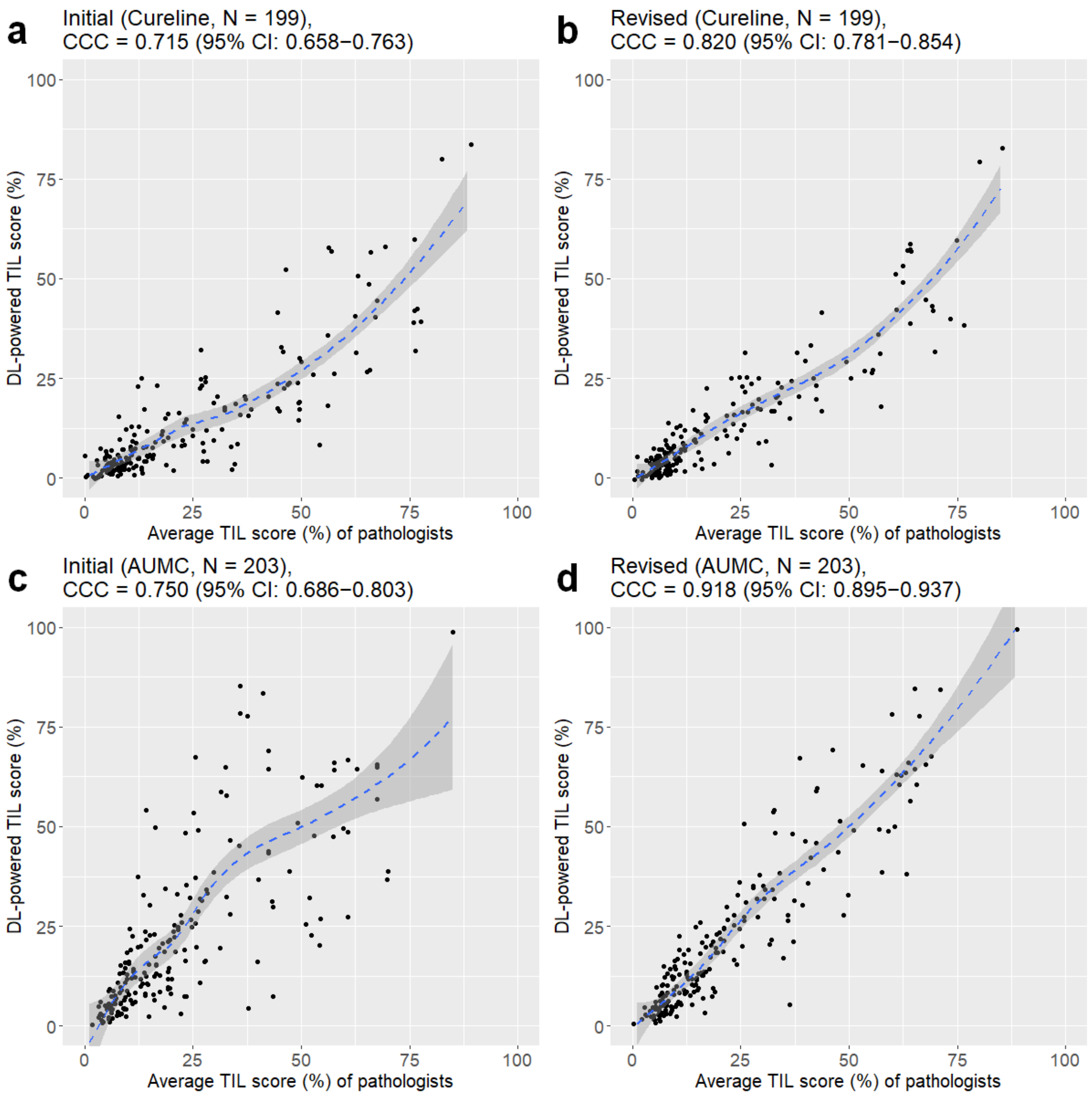

Supplementary Fig. 4. (**a-b**) The concordance correlation coefficient (CCC) values between the average sTIL score among pathologist and the deep learning (DL) interpretation in the Cureline set before (**a**) and after DL assistance (**b**). (**c-d**) The CCC values between the average sTIL score among pathologist and the DL interpretation in the Ajou University Medical Center (AUMC) set before (**c**) and after DL assistance (**d**).

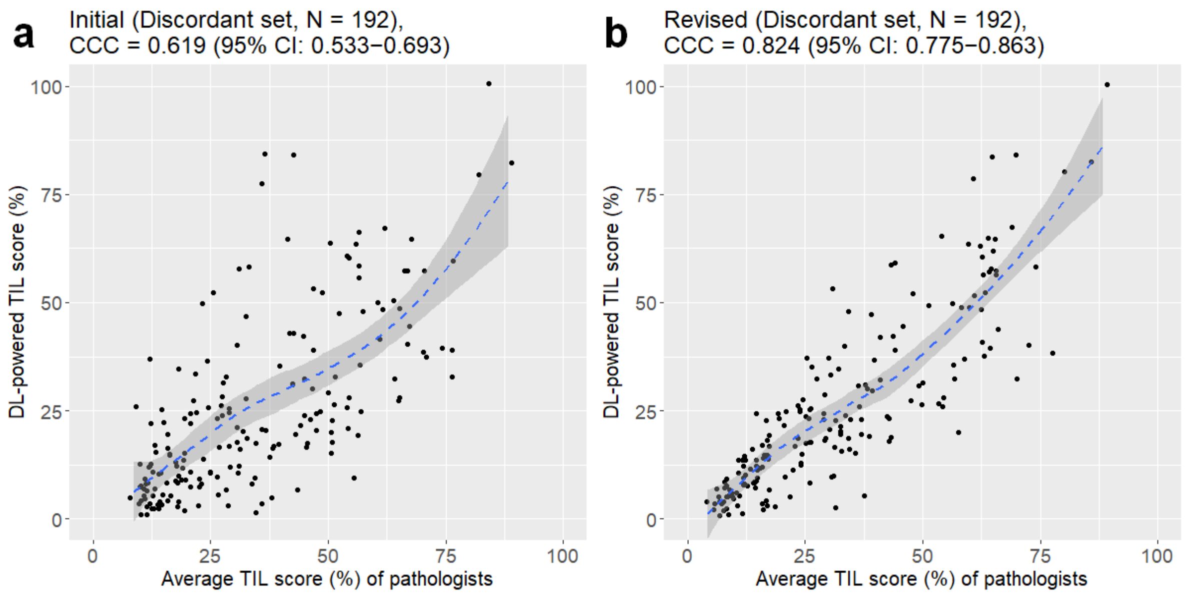

Supplementary Fig. 5. The concordance correlation coefficient (CCC) values between the average stromal tumor infiltrating lymphocyte (sTIL) score among pathologist and the deep learning (DL) interpretation in the initially discordant set ($N = 169$). (**a**) Before DL assistance (**b**) After DL assistance.

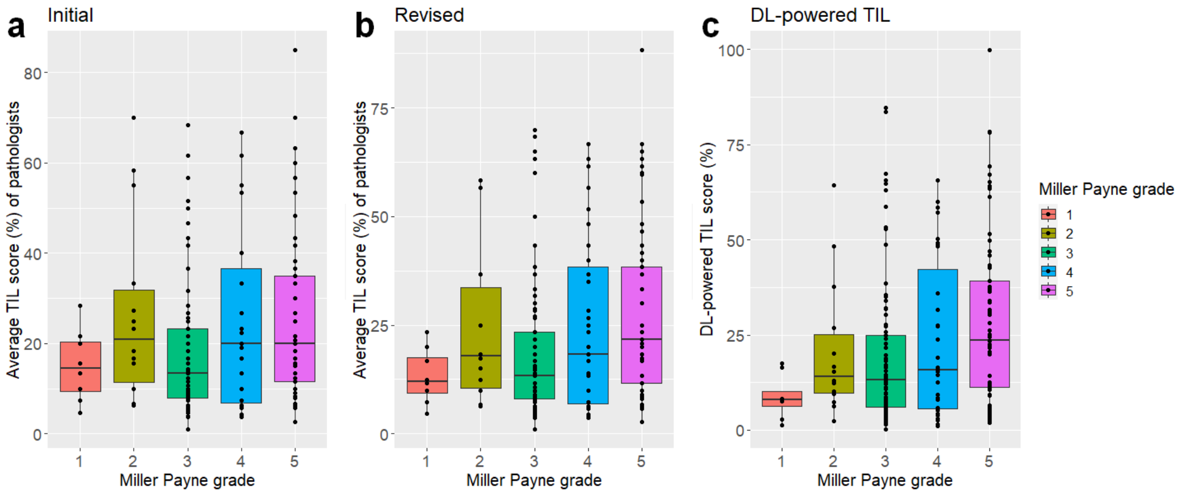

Supplementary Fig. 6. The average stromal tumor infiltrating lymphocyte (sTIL) score in the Miller-Payne grade subgroup evaluated by (**a**) initial consensus among pathologists, (**b**) deep learning (DL)-assisted revision consensus among pathologists, and (**c**) the DL model. The center lines in the boxplot represent median values; the bounds of the boxplot represent the interquartile ranges; the whiskers represent the range of the data.

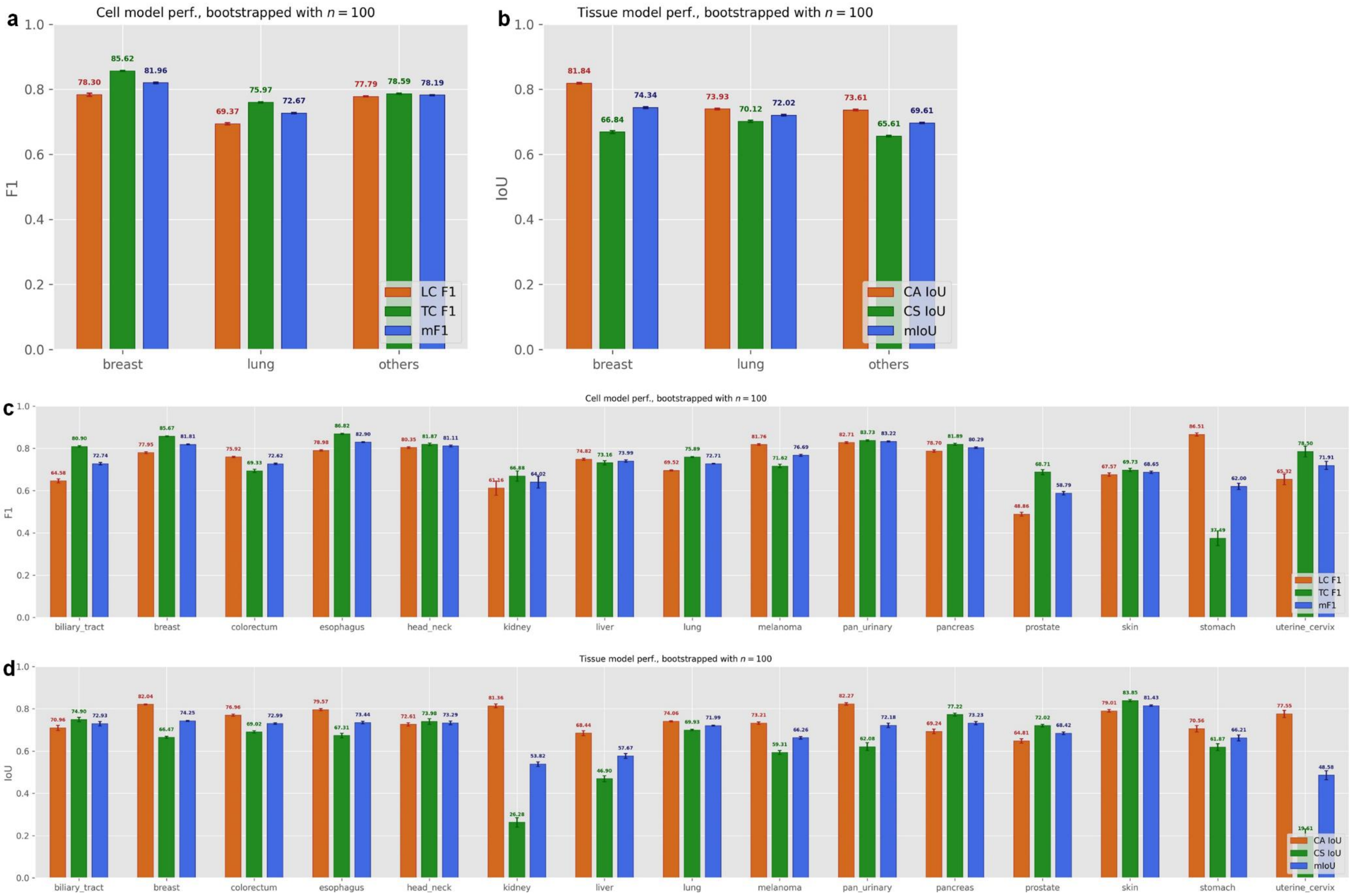
a
Cell model perf., bootstrapped with $n = 100$
F1
1.0
0.8
0.6
0.4
0.2
0.0
78.30
85.62
81.96
69.37
75.97
72.67
77.79
78.59
78.19
breast
lung
others
LC F1
TC F1
mF1
b
Tissue model perf., bootstrapped with $n = 100$
IoU
81.84
66.84
74.34
73.93
70.12
72.02
73.61
65.61
69.61
breast
lung
others
CA IoU
CS IoU
mIoU
c
Cell model perf., bootstrapped with $n = 100$
F1
64.58 80.90 72.74
77.95 85.67 81.81
75.92 69.33 72.62
78.98 86.82 82.90
80.35 81.87 81.11
61.16 66.88 64.02
74.82 73.16 73.99
69.52 75.89 72.71
81.76 71.62 76.69
82.71 83.73 83.22
78.70 81.89 80.29
48.86 68.71 58.79
67.57 69.73 68.65
86.51 37.49 62.00
65.32 78.50 71.91
biliary_tract
breast
colorectum
esophagus
head_neck
kidney
liver
lung
melanoma
pan_urinary
pancreas
prostate
skin
stomach
uterine_cervix
LC F1
TC F1
mF1
d
Tissue model perf., bootstrapped with $n = 100$
IoU
70.96 74.90 72.93
82.04 66.47 74.25
76.96 69.02 72.99
79.57 67.31 73.44
72.61 73.98 73.29
81.36 26.28 53.82
68.44 46.90 57.67
74.06 69.93 71.99
73.21 59.31 66.26
82.27 62.08 72.18
69.24 77.22 73.23
64.81 72.02 68.42
79.01 83.85 81.43
70.56 61.87 66.21
77.55 19.61 48.58
biliary_tract
breast
colorectum
esophagus
head_neck
kidney
liver
lung
melanoma
pan_urinary
pancreas
prostate
skin
stomach
uterine_cervix
CA IoU
CS IoU
mIoU

Supplementary Fig. 7. A bootstrapping evaluation result of deep learning-based tumor infiltrating lymphocyte analyzer on the cell (**a**) and the tissue (**b**) of breast cancers, lung cancers, and other cancer types. (**c, d**) The cell and tissue model performance of the DL model in the various cancer types analyzed. The error bars represent 95% confidence intervals. The error bars are 95% confidence intervals.

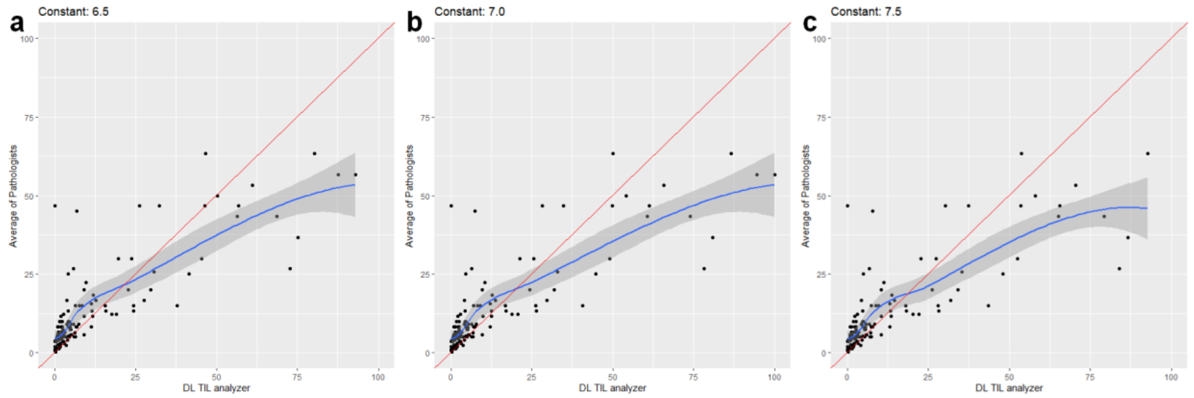

Supplementary Fig. 8. Correlation between the deep learning (DL)-based tumor infiltrating lymphocyte (TIL) analyzer and the average stromal TIL of pathologists according to variations in constant α value (**a**: Constant 6.5, **b**: Constant 7.0, **c**: Constant 7.5).

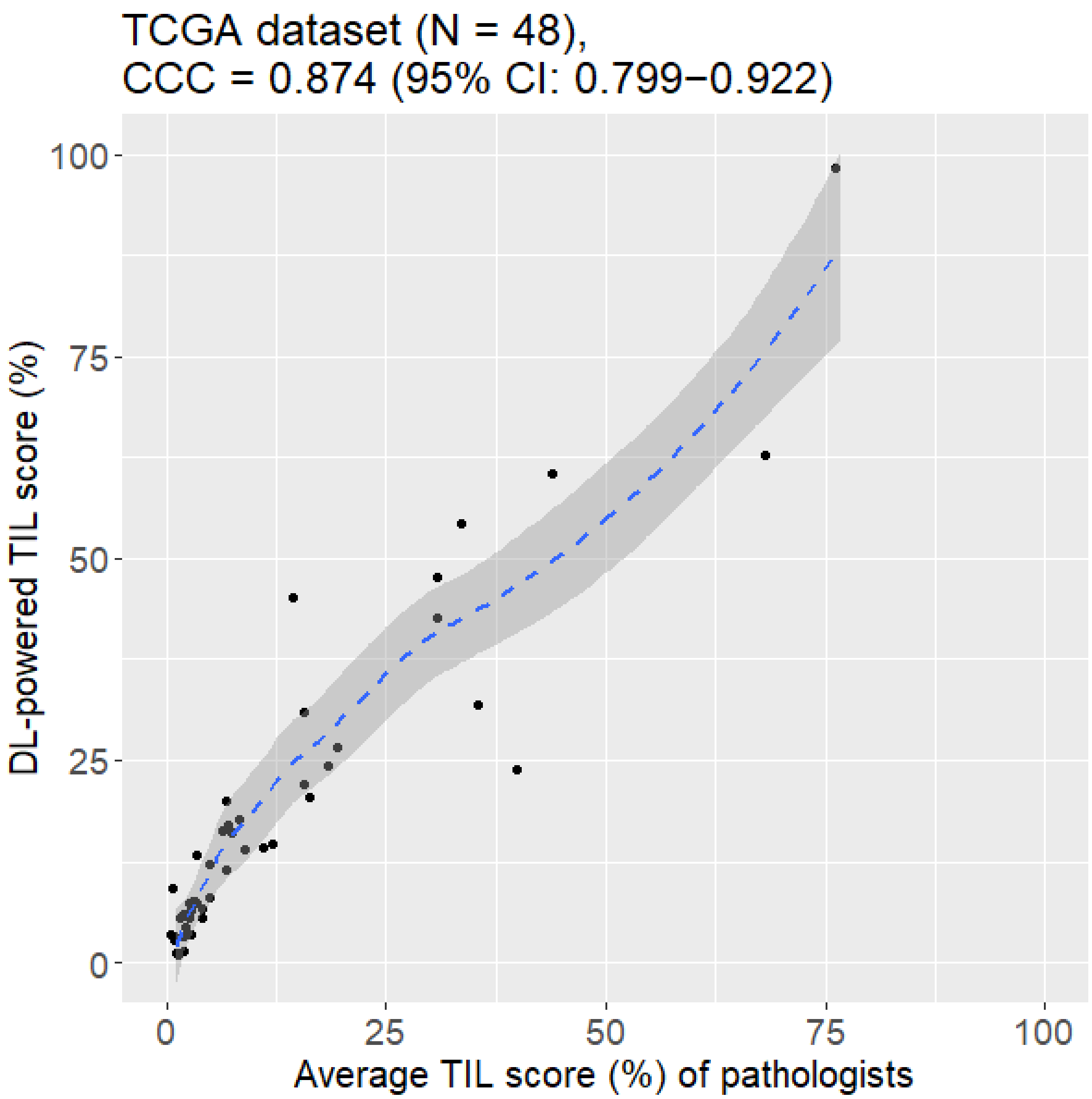

Supplementary Fig. 9. Correlation between the deep learning (DL)-based tumor infiltrating lymphocyte (TIL) analyzer and the average stromal TIL of pathologists in TCGA validation dataset (*N* = 48), measured by concordance correlation coefficient (CCC).
