# Supplementary material for: Deep learning model improves tumor-infiltrating lymphocyte evaluation and therapeutic response prediction in breast cancer
Source: NPJ Breast Cancer. 2023 Aug 30;9:71. doi: 10.1038/s41523-023-00577-4 (PMC10469174; doi:10.1038/s41523-023-00577-4)
Supplement: Supplementary file 2 — Reporting summary [file 41523_2023_577_MOESM2_ESM.pdf]

nature portfolio

Corresponding author(s): Seokhwi Kim

Last updated by author(s): Aug 11, 2023

Reporting Summary

Nature Portfolio wishes to improve the reproducibility of the work that we publish. This form provides structure for consistency and transparency in reporting. For further information on Nature Portfolio policies, see our Editorial Policies and the Editorial Policy Checklist.

Statistics

For all statistical analyses, confirm that the following items are present in the figure legend, table legend, main text, or Methods section.

n/a	Confirmed	
☐	☒	The exact sample size (*n*) for each experimental group/condition, given as a discrete number and unit of measurement
☐	☒	A statement on whether measurements were taken from distinct samples or whether the same sample was measured repeatedly
☐	☒	The statistical test(s) used AND whether they are one- or two-sided<br>*Only common tests should be described solely by name; describe more complex techniques in the Methods section.*
☒	☐	A description of all covariates tested
☐	☒	A description of any assumptions or corrections, such as tests of normality and adjustment for multiple comparisons
☐	☒	A full description of the statistical parameters including central tendency (e.g. means) or other basic estimates (e.g. regression coefficient) AND variation (e.g. standard deviation) or associated estimates of uncertainty (e.g. confidence intervals)
☐	☒	For null hypothesis testing, the test statistic (e.g. *F*, *t*, *r*) with confidence intervals, effect sizes, degrees of freedom and *P* value noted<br>*Give P values as exact values whenever suitable.*
☒	☐	For Bayesian analysis, information on the choice of priors and Markov chain Monte Carlo settings
☒	☐	For hierarchical and complex designs, identification of the appropriate level for tests and full reporting of outcomes
☐	☒	Estimates of effect sizes (e.g. Cohen's *d*, Pearson's *r*), indicating how they were calculated

Our web collection on statistics for biologists contains articles on many of the points above.

Software and code

Policy information about availability of computer code

Data collection	The software was developed using Python programming language (version 3.7). The models are implemented using PyTorch v1.4 (available at https://github.com/pytorch/pytorch). Clinical dataset was collected using R software v4.0.3.
Data analysis	The software was developed using Python programming language (version 3.7). The models are implemented using PyTorch v1.4 (available at https://github.com/pytorch/pytorch). The cell detection model is based on a proprietary implementation of Faster R-CNN (open source implementations available online, e.g., at https://github.com/pytorch/vision/blob/main/torchvision/models/detection/faster_rcnn.py), with a ResNet-34 backbone architecture pre-trained on ImageNet data (pre-trained weights available at https://download.pytorch.org/models/resnet34-333f7ec4.pth and open-source implementation at https://github.com/pytorch/vision/blob/master/torchvision/models/resnet.py). The tissue segmentation model is based on a proprietary implementation of DeepLabV3 (open source implementations available online, e.g., at https://github.com/VainF/DeepLabV3Plus-Pytorch), with an EfficientNet-B3 backbone architecture pre-trained on ImageNet data (pre-trained weights available at http://storage.googleapis.com/public-models/efficientnet-b3-c8376fa2.pth and open-source implementation at https://github.com/lukemelas/EfficientNet-PyTorch). The data augmentation transformations and image manipulation routines are implemented using TorchVision v0.5.0 (https://github.com/pytorch/vision), Albumentations v0.4.5 (https://github.com/albumentations-team/albumentations), OpenCV Python v4.1.2.30 (https://github.com/opencv/opencv-python), and Scikit-image v0.16.2 (https://github.com/scikit-image/scikit-image). Mathematical and statistical operations are implemented using Numpy v1.18.1 (https://github.com/numpy/numpy), Pandas v1.0.1 (https://github.com/pandas-dev/pandas), Scipy v1.3.0 (https://github.com/scipy/scipy), and Scikit-learn v0.22.1 (https://github.com/scikit-learn/scikit-learn). Finally, the WSIs were read and manipulated using OpenSlide v3.4.1 (https://github.com/openslide/openslide) and corresponding Pyhton wrapper v1.1.1 (https://github.com/openslide/openslide-python). Statistical analysis was conducted using R software v4.0.3. The R code and csv file to create the figures can be found at https://github.com/SooIckCho/Breast_TIL.

For manuscripts utilizing custom algorithms or software that are central to the research but not yet described in published literature, software must be made available to editors and reviewers. We strongly encourage code deposition in a community repository (e.g. GitHub). See the Nature Portfolio guidelines for submitting code & software for further information.

Data

Policy information about availability of data

All manuscripts must include a data availability statement. This statement should provide the following information, where applicable:

- Accession codes, unique identifiers, or web links for publicly available datasets
- A description of any restrictions on data availability
- For clinical datasets or third party data, please ensure that the statement adheres to our policy

The pathologists' evaluation results and the inference results of the DL-powered model on 171 grids and 48 WSIs from TCGA can be accessed from the following link: https://drive.google.com/file/d/1aVdRHpjGDMWE3n_TH07ZiG8nzA-w9YZt/view?usp=share_link. The visualization and validation of the algorithm is available from the DL-powered model page which is available upon request. The other data (Cureline and AUMC dataset) in this study are also available from the corresponding author upon academic purpose-request.

Research involving human participants, their data, or biological material

Policy information about studies with human participants or human data. See also policy information about sex, gender (identity/presentation), and sexual orientation and race, ethnicity and racism.

Reporting on sex and gender	All specimens from Cureline were surgically resected primary breast cancer from treatment-naïve female patients.
Reporting on race, ethnicity, or other socially relevant groupings	In Table 1, ethnicity is described as "White patients" and "Asian patients".
Population characteristics	Table 1 describes the age, molecular subtype of breast cancer, histologic subtype, Bloom-Richardson grade, Black's nuclear grade, and cancer stage.
Recruitment	This study was a retrospective analysis of clinical data from eligible subjects at specific institutions.
Ethics oversight	The data were collected and utilized under the permission of the Institutional Review Boards (IRBs). For the Cureline dataset, the IRB from Saint Petersburg City Clinical Oncology Hospital approved the study under the protocol CU-2010 Oncology 12152009, and the IRB from Saint Petersburg Academic University of the Russian Academy of Sciences approved protocol number CU-M-07092015-C-INT. For the AUMC dataset, the IRB from Ajou University Medical Center approved the study under the protocol AJOUIRB-KS-2022-340. Informed consent was waived by the IRB because of the retrospective nature of the study and the anonymized clinical data used in the analysis.

Note that full information on the approval of the study protocol must also be provided in the manuscript.

Field-specific reporting

Please select the one below that is the best fit for your research. If you are not sure, read the appropriate sections before making your selection.

☒ Life sciences ☐ Behavioural & social sciences ☐ Ecological, evolutionary & environmental sciences

For a reference copy of the document with all sections, see nature.com/documents/nr-reporting-summary-flat.pdf

Life sciences study design

All studies must disclose on these points even when the disclosure is negative.

Sample size	This study was retrospective and was conducted at a specific institution with a sample of subjects who met certain criteria. A total of 402 cases were included, of which 199 and 203 were analyzed from the two types of data sources, respectively, which can be considered sufficient for a general medical study.
Data exclusions	This study excluded cases that did not meet the data collection criteria.
Replication	Not applicable
Randomization	Not applicable
Blinding	Not applicable

Reporting for specific materials, systems and methods

We require information from authors about some types of materials, experimental systems and methods used in many studies. Here, indicate whether each material, system or method listed is relevant to your study. If you are not sure if a list item applies to your research, read the appropriate section before selecting a response.

Materials & experimental systems

n/a	Involved in the study
☒	☐ Antibodies
☒	☐ Eukaryotic cell lines
☒	☐ Palaeontology and archaeology
☒	☐ Animals and other organisms
☒	☐ Clinical data
☒	☐ Dual use research of concern
☒	☐ Plants

Methods

n/a	Involved in the study
☒	☐ ChIP-seq
☒	☐ Flow cytometry
☒	☐ MRI-based neuroimaging
